# Treatment of Glaucoma with Natural Products and Their Mechanism of Action: An Update

**DOI:** 10.3390/nu14030534

**Published:** 2022-01-26

**Authors:** Ru Hui Sim, Srinivasa Rao Sirasanagandla, Srijit Das, Seong Lin Teoh

**Affiliations:** 1 Tanglin Health Clinic, Kuala Lumpur 50480, Malaysia; simruhui@gmail.com; 2 Department of Human & Clinical Anatomy, College of Medicine & Health Sciences, Sultan Qaboos University, Al-Khoud, Muscat 123, Oman; srinivasa@squ.edu.om; 3 Department of Anatomy, Faculty of Medicine, Universiti Kebangsaan Malaysia Medical Centre, Cheras, Kuala Lumpur 56000, Malaysia

**Keywords:** glaucoma, herbs, traditional medicine, retinal ganglion cells, intraocular pressure

## Abstract

Glaucoma is one of the leading causes of irreversible blindness. It is generally caused by increased intraocular pressure, which results in damage of the optic nerve and retinal ganglion cells, ultimately leading to visual field dysfunction. However, even with the use of intraocular pressure-lowering eye drops, the disease still progresses in some patients. In addition to mechanical and vascular dysfunctions of the eye, oxidative stress, neuroinflammation and excitotoxicity have also been implicated in the pathogenesis of glaucoma. Hence, the use of natural products with antioxidant and anti-inflammatory properties may represent an alternative approach for glaucoma treatment. The present review highlights recent preclinical and clinical studies on various natural products shown to possess neuroprotective properties for retinal ganglion cells, which thereby may be effective in the treatment of glaucoma. Intraocular pressure can be reduced by baicalein, forskolin, marijuana, ginsenoside, resveratrol and hesperidin. Alternatively, *Ginkgo biloba*, *Lycium barbarum*, *Diospyros kaki*, *Tripterygium wilfordii*, saffron, curcumin, caffeine, anthocyanin, coenzyme Q10 and vitamins B3 and D have shown neuroprotective effects on retinal ganglion cells via various mechanisms, especially antioxidant, anti-inflammatory and anti-apoptosis mechanisms. Extensive studies are still required in the future to ensure natural products’ efficacy and safety to serve as an alternative therapy for glaucoma.

## 1. Introduction

Glaucoma is one of the leading causes of irreversible blindness, causing 6.6% of all blindness in 2010 [1]. According to the World Health Organization’s (WHO) World Report on Vision, of the estimated 2.2 billion people having a vision impairment around the world, glaucoma affects an estimated 6.9 million people [2]. It has been further estimated that by 2040, approximately 111.8 million people worldwide aged between 40 and 80 years old will be affected by glaucoma [3]. Glaucoma is generally caused by intraocular pressure (IOP, >21 mmHg) build-up, resulting from blockage of intraocular fluid and aqueous humor drainage [4]. The elevated IOP progressively damages the retinal ganglion cells (RGCs) and optic nerve, causing visual field constriction that affects the peripheral field initially and the central vision field gradually [5]. Glaucoma patients require lifelong treatment and follow-up, and the disease has a significant negative impact on patients’ quality of life in terms of anxiety, psychological well-being, daily life, driving and confidence in healthcare [6]. The main risk factors for glaucoma prevalence include age, family history with glaucoma, African American race, thinner central corneal thickness, pseudoexfoliation, pigment dispersion and myopia [7]. Additionally, an association between diabetes, hypertension, triglyceride levels and glaucoma were also identified [7,8]. Furthermore, genetic factors are also known to be risk factors for glaucoma, in which single-nucleotide polymorphisms in numerous genes (e.g., myocilin, apolipoprotein E, X-ray repair cross-complementing group 1, zona pellucida glycoprotein 4) have been shown to be associated with an increased risk of glaucoma [9,10].

Glaucoma can be classified into two major types, i.e., open-angle (OAG) and angle-closure glaucoma (ACG), according to the physical obstruction of the aqueous humor drainage system, and the appearance of the iridocorneal angle and trabecular meshwork (TM) [11]. Alternatively, it can also be categorized as primary (idiopathic, not associated with other diseases or conditions) or secondary (attributed to underlying diseases or conditions, such as trauma, long-term medication, ophthalmic surgery, uveitis, necrotic tumors, diabetes or syndromic conditions) [11,12].

In primary OAG (POAG), aqueous humor drainage is obstructed or inadequate as there is an internal blockage within the TM [13]. In contrast, primary ACG (PACG) is characterized by the presence of a physical obstacle to the aqueous drainage as the iris is adhered to the cornea, obstructing the flow of aqueous humor to the TM and the uveoscleral drainage [12,14]. Symptoms appear more drastically in PACG, which results in a rapid reduction in the vision field, leading to total blindness. Other symptoms include ocular pain, headache, nausea, vomiting, multicolored halos and blurred vision [12]. Additionally, PACG is an ophthalmic emergency that requires immediate treatment to prevent the progression of irreversible ocular damage [12].

## 2. Pathogenesis of Glaucoma

The exact pathogenesis of glaucoma is complex and has not yet been fully elucidated. The potential mechanism involved in the neurodegeneration of glaucoma has been postulated to involve an amalgamation of mechanical, vascular, genetic and immunological factors.

### 2.1. Mechanical Hypothesis

The mechanical hypothesis explains the relationship between the IOP and RGC pathophysiology. The perforated lamina cribrosa (LC) is the weakest part of the sclera, and it is where the RGC axons pierce through the minute perforations to form the optic nerve, while the central retinal artery and vein pass through the LC via a larger central aperture [15]. Elevated IOP resulted from the imbalance between the production and drainage of aqueous humor, which led to the irreversible backwards bowing of the LC, in the process known as ‘cupping’ [16]. Optic nerve cupping is characterized by the remodeling of the extracellular matrix (ECM) and fibrosis in the LC [17]. Glaucomatous LC cells showed increased ECM gene expression and elevated intracellular calcium, which is known to promote proliferation, activation and contractility in fibroblasts via the nuclear factor of activated T cells/calcium signaling pathway [17]. This deformation damages the optic nerve and capillaries passing through the LC, disturbing the anterograde axonal transportation of RGCs, which then ultimately triggers visual field defects in glaucoma [16]. Furthermore, elevated IOP also resulted in activated pro-fibrotic pathway-induced ECM accumulation in the TM, leading to less efficient aqueous humor outflow, thereby causing further damage to the LC [18].

Ivers et al. [19] demonstrated that in experimental glaucoma monkeys, the first structural abnormality induced by elevated IOP was an increased anterior LC surface depth, followed by a decreased minimum rim width, and, lastly, a reduced retinal nerve fiber layer (RNFL) thickness. Different levels of increased IOP showed a remarkable effect on the visual field, best-corrected visual acuity and LC parameters (cup depth, LC depth, LC curvature index and prelaminar tissue thickness) [20]. Additionally, greater posterior displacement of the LC was significantly associated with a faster rate of loss of the RNFL [21]. RGC axonal degeneration and anterograde axonal transport deficits at the optic nerve head (ONH, the location where RGC axons converge to form the optic nerve and traverse the LC) precede the structural and functional loss of RGCs [22]. Disturbance of the RGC anterograde axonal transport leads to the accumulation of metabolic waste in the cells and deprives the metabolic needs of the RGCs, subsequently causing their apoptosis [23].

In normal-tension glaucoma (NTG), patients also present with glaucomatous optic disc excavation, despite a normal IOP [24]. This suggests other risk factors are involved in the optic neurodegeneration of glaucoma. The LC serves as a barrier between the IOP within the eye, and the intracranial pressure within the cerebrospinal fluid-filled subarachnoid space surrounding the optic nerve; the pressure gradient between the LC is known as the translaminar pressure gradient (TLPG) [25,26]. The TLPG is higher in glaucoma patients, including NTG patients, and is associated with mechanical damage to the optic nerve fibers, anterograde axonal transportation disruption and altered blood flow, leading to glaucomatous damage [26,27,28].

### 2.2. Vascular Hypothesis

The blood flow of the ONH was significantly reduced in the eyes of pre-perimetric glaucoma patients, where there are characteristic glaucomatous changes in the optic disc, but without the presence of visual field defects [29,30]. POAG and PACG patients possess a lower capillary density, but with greater tortuosity and more dilated capillaries, compared to healthy individuals [31]. Similarly, both NTG and POAG patients showed lower retrobulbar velocities, and higher retinal venous saturation and choroidal thickness asymmetries, when compared to control subjects [32]. Decreased ocular blood flow was also shown to be correlated with structural glaucomatous progression, as indicated by retinal and optic nerve changes [33]. A recent retrospective longitudinal study revealed that reduced blood flow in the ONH precedes glaucomatous neurodegeneration in POAG patients [34]. The vascular hypothesis is thus based on the reduced perfusion pressure, faulty vascular autoregulation or loss of neurovascular coupling, which leads to optic nerve degeneration in glaucoma [35]. Due to the reduced ocular blood flow, this hypothesis proposes that the RGC axons suffer from oxygen and nutrient insufficiency, ultimately causing their degeneration. In a glaucoma rat model, ocular hypertension (OHT) led to selective hypoxia in the LC, which was associated with injured RGC axons, and axonal transport disruption [36]. This study also demonstrated upregulation of hypoxia-inducible enzyme heme oxygenase-1 (HO-1) and the anaerobic glycolytic enzyme lactate dehydrogenase, and increased generation of superoxide radicals in the retina and ONH, as well as the active subunit of the superoxide-generating enzyme NADPH oxidase, suggesting the involvement of oxidative stress [36]. Similarly, hypoxic RGCs were observed in young and aged glaucoma model DBA/2J (D2) mouse retinas, with a significant increase in the hypoxia-inducible factor-1α (HIF-1α) protein and reactive oxygen species (ROS), followed by a significant decrease in the antioxidant capacity and mitochondrial mass in the aged retinas [37].

### 2.3. Oxidative Stress and Neuroinflammation in Glaucoma

In accordance with animal studies, numerous studies have provided evidence of increased oxidative stress in glaucoma patients. In addition, blood and aqueous humor levels of oxidative stress-related molecular biomarkers, i.e., protein carbonyls and advanced glycation end products, significantly increased in glaucomatous samples compared with healthy controls [38]. Similarly, PACG patients presented with decreased serum levels of total antioxidant status (TAS) and superoxide dismutase (SOD), as well as increased levels of malondialdehyde (MDA), compared to healthy controls [39]. A meta-analysis further indicated that POAG patients had lower TAS in the blood and higher levels of SOD, glutathione peroxidase (GPX) and catalase (CAT) in the aqueous humor [40]. Oxidative stress is known to induce or dysregulate inflammation in the event of optic neurodegeneration from glaucoma.

Studies have shown that inflammation contributes to the disease progression of glaucoma. In glaucomatous human optic nerves, the number of CD163+ cells (a commonly used marker for anti-inflammatory macrophages involved in tissue repair and remodeling) was significantly increased [41]. Systemic inflammatory status markers, i.e., the neutrophil-to-lymphocyte ratio, platelet-to-lymphocyte ratio and systemic immune inflammation index, were significantly increased in POAG patients compared with the control group [42]. POAG patients exhibited a significant increase in various cytokines, i.e., serum interleukin (IL)-4, -6 and -12p70 and tumor necrosis factor-alpha (TNF-α), compared with the controls [43]. Similarly, elevated plasma TNF-α levels in patients with POAG and pseudoexfoliation glaucoma were detected [44,45]. Additionally, aqueous humor samples collected from chronic PACG patients showed significantly increased levels of eotaxin, macrophage inflammatory protein-1-alpha and interferon gamma (IFN-γ)-induced protein-10, and lower levels of TNF-α, IL-5, -9 and -17 and granulocyte-macrophage colony-stimulating factor, compared to the control group [46].

Glial cells in the retina, i.e., astrocytes, Müller cells and microglial cells having an important role in mediating inflammatory responses, have been shown to become reactive, leading to the production of inflammatory cytokines, causing further neuronal damage in glaucoma patients and experimental glaucoma models [47,48]. In general, cytokine signaling is linked to the inflammatory transducer nuclear factor-kappa B (NF-κB). In D2 mice, low energy-induced 5′ adenosine monophosphate-activated protein kinase (AMPK) phosphorylation in the retina and optic nerve triggered NF-κB p65 signaling, leading to increased pro-inflammatory TNF-α, IL-6 and nitric oxide synthase (NOS)-2 expression [49]. Injection administration of TGF-β2 increased IOP and ECM deposition in the TM of wild-type mice. In contrast, mice harboring a mutation in NF-κB blocked the effect, suggesting NF-κB is necessary for TGF-β2-induced ECM production and OHT [50]. Additionally, transgenic inhibition of astroglial NF-κB restrained the neuroinflammatory (reduced pro-inflammatory cytokine expressions, i.e., IL-1A, -1B, -2, -6, -10, -12 and -13, TNF-α and IFN-γ) and neurodegenerative outcomes (attenuated loss of RGCs and axons) of the eyes of an experimental OHT mouse model [51].

The current evidence indeed supports the contribution of neuroinflammation in the pathogenesis of glaucoma, but it is still not clear as to when neuroinflammation takes part in the sequence of pathological events in glaucoma. Neuroinflammation has been suggested to be secondary to the initial pathology (i.e., optic nerve crush injury) [52]. Optic nerve crush injury induced glial activation in the retina, which was significantly muted if RGC death was blocked by deletion of the Bax gene [52]. On the other hand, the inhibition of monocyte infiltration and microglial activation by X-ray treatment prevented neuronal damage and dysfunction in the ONH [53]. Nevertheless, immunomodulation has been shown to be beneficial in the progression of glaucomatous changes.

### 2.4. Excitotoxicity of Glutamate

In addition to the inflammatory response, glial cells in the retina also play a vital role in the function of the retina by providing homeostatic and metabolic support to the photoreceptors and retinal neurons [54]. Müller cells and astrocytes possess uptake and exchange systems for various neurotransmitters, including glutamate, via the glutamate/aspartate transporter (GLAST) in rodents, also known as the Na^+^-dependent high-affinity glutamate transporter-1 (EAAT-1) in humans [54,55]. Glaucomatous eyes have been shown to have decreased levels of EAAT-1, and the glutamate receptor subunit *N*-methyl-d-aspartate (NMDA)-R1 [56]. Furthermore, mice deficient in GLAST demonstrate spontaneous RGC loss and optic nerve degeneration without elevated IOP, suggesting the decrease in GLAST expression leads to glutamate excitotoxicity in the retina, as a possible pathogenesis of glaucoma [57].

**Figure 1:**
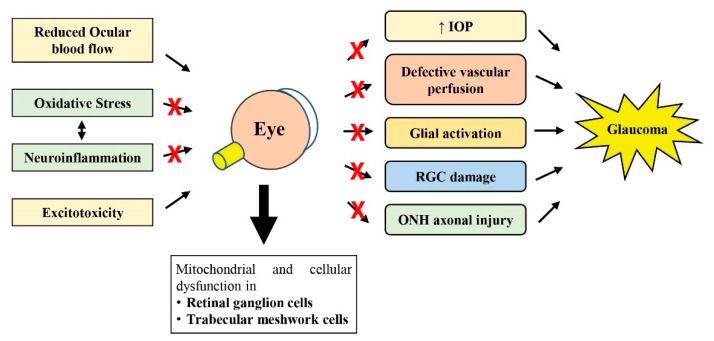
Schematic diagram showing how oxidative stress, neuroinflammation, reduced ocular blood flow and excitotoxicity lead to subsequent pathological changes observed in glaucoma. The therapeutic potential of natural products against glaucomatous changes at various steps is shown with the symbol ×. RGC, retinal ganglion cell; IOP, intraocular pressure; ONH, optic nerve head.

As reviewed by others, perhaps the most accepted hypothesis involved in glaucoma pathogenesis currently may include the mechanical damage to the ONH induced by increased IOP, followed by vascular dysregulation (reduced ocular blood flow) and neuroinflammation (glial activation), which then disrupt axonal transport due to axonal mitochondrial function loss in the RGCs, ultimately leading to RGC axonal degeneration and RGC cell death (Figure 1) [58,59,60]. However, the combination of mechanisms described earlier may vary greatly among different glaucoma patients [60].

## 3. Glaucoma Research Models

Numerous research models have been used to gain a considerable understanding of the pathogenesis of glaucoma, and to assess therapeutic approaches for glaucoma treatments [61,62,63,64]. In this section, we provide a brief overview of some of these models used by the studies presented in this review (summarized in Table 1); this helps to provide a better understanding of the discussions in the following sections.

There are several genetic glaucomatous animal models that present with an elevated or normal IOP. For instance, the D2 mouse presents a late-onset, chronic pigmentary glaucoma due to the high IOP that progresses with age, resulting from tyrosinase-related protein 1 (*Tyrp1*) mutation and a premature stop codon in glycoprotein non-metastatic melanoma protein B (*Gpnmb*), which collectively lead to anterior segment anomalies, iris atrophy, peripheral anterior synechiae and pigment dispersion [64,65]. In contrast, D2-*Gpnmb*^+^ mice are the wild types for the *Gpnmb* mutation that do not develop increased IOP and glaucoma [66]. Alternatively, the *Vav2*/*Vav3*-deficient and connective tissue growth factor (βB1-CTGF) mouse models are other murine models of spontaneous glaucoma that present with elevated IOP, which leads to subsequent RGC loss [67,68]. Transgenic mice with a low overexpression of E50K mutant optineurin (E50K-OPTN) have been reported to present with enhanced axonal degeneration and decreased RGC survival, under normal IOP [69].

Glaucoma can also be induced in wild-type animal models by elevating the IOP experimentally. A high IOP can be achieved by blocking aqueous humor drainage with the injection of various substances (e.g., microbeads, hydroxypropyl methylcellulose and hyaluronic acid) into the anterior chamber [70,71,72]. Alternatively, injection of hypertonic saline into the episcleral vein [73], and cauterization [74] or laser photocoagulation [75,76,77] of the episcleral or limbal veins lead to TM scarring, which increases the resistance to aqueous humor drainage, resulting in an elevation in IOP. The elevated IOP in these models leads to varying degrees of RGC loss, glial activation and visual defects [75,76,77,78].

To investigate the role of excitotoxicity in glaucoma, RGC loss can be induced with the injection of NMDA intravitreally [79]. The optic nerve crush (performed by applying a crush injury to the optic nerve with a pair of cross-action forceps) or the complete optic nerve transection model causes all RGC axons to be damaged simultaneously, which results in the gradual loss of RGCs [80,81]. This non-IOP-related axonal degeneration research model is commonly used to assess the RGC neuroprotection properties of various substances [82]. The partial optic nerve transection model causes damage to only a portion of the RGC axons; thus, this model can study both primary (the death of RGCs whose axons have been cut off) and delayed secondary neurodegeneration (the death of RGCs whose axons are intact) [83]. Retinal ischemia/reperfusion (I/R) injury is known to be associated with glaucoma, and other eye diseases, and has been widely used as an animal model for OAG. I/R injury reduces retinal blood flow, which creates a state of retinal hypersensitivity to oxygen and other nutrients, precipitating severe oxidative and inflammatory damage when the circulation is subsequently reinstated (reperfusion) [84,85].

**Table 1:** Overview of glaucoma research models.

Research Models		Genes Involved	Mechanisms	References
Genetic in vivo model	D2 mice	Tyrosinase-related protein 1 (*Tyrp1*) Glycoprotein non-metastatic melanoma protein B (*Gpnmb*)	Blockage of aqueous humor drainage, leading to progressive elevated IOP	[65]
	Methods	Surgery involved	Mechanisms	References
Experimental in vivo model	Injection	Injection of microbeads into the anterior chamber	Blockage of aqueous humor drainage, leading to elevated IOP	[70]
		Injection of hydroxypropyl methylcellulose into the anterior chamber	Blockage of aqueous humor drainage, leading to elevated IOP	[71]
		Injection of hyaluronic acid into the anterior chamber	Blockage of aqueous humor drainage, leading to elevated IOP	[72]
		Injection of hypertonic saline into the episcleral vein	Produced scarring in the TM, increasing resistance to aqueous humor drainage, leading to elevated IOP	[73]
		Intravitreal injection of NMDA	NMDA induced excitotoxicity, leading to RGC death	[79]
	Cauterization/laser photocoagulation	Episcleral vein cauterization	Produced scarring in the TM, increasing resistance to aqueous humor drainage, leading to elevated IOP	[74]
		Argon laser photocoagulation of the episcleral/limbal vein	Produced scarring in the TM, increasing resistance to aqueous humor drainage, leading to elevated IOP	
	Nerve injury	Optic nerve crush	Optic nerve injury leading to axonal degeneration and gradual RGC loss	[80]
		Complete optic nerve transection	Optic nerve injury leading to axonal degeneration and gradual RGC loss	[81]
		Partial optic nerve transection	Optic nerve injury leading to axonal degeneration and gradual RGC loss	[83]
	Retinal I/R injury	Reduced retinal blood flow by induction of elevated IOP (ischemia), followed by reinstation of blood flow (reperfusion)	Extreme acute OHT-induced ischemic injury to RGC, followed by severe oxidative and inflammatory damage to RGCs after reperfusion	[84,85]

D2, DBA/2J; I/R, ischemia/reperfusion; IOP, intraocular pressure; NMDA, *N*-methyl-d-aspartate; RGC, retinal ganglion cell.

Numerous in vitro studies have utilized the RGC-5 cell line in glaucoma research to evaluate the neuroprotective properties of various supplements, including the studies reviewed here. However, it has now become clear that RGC-5 cells that were originally identified as immortalized rat RGCs were contaminated early in their development by the immortalized photoreceptor 661W cell line (RGC precursor-like cells) in the laboratory they originated from [86,87]. Therefore, the RGC-5 cells used by many of the studies described in the following section may not reflect the true phenotype of a mature RGC. Perhaps the use of primary RGCs from animal models would be better to investigate glaucomatous RGC responses to therapies in vitro [88].

In general, the various research models described represent only some aspects of glaucoma, thus each having different advantages over other models. It is important to use a suitable model based on the objective of the study.

## 4. Natural Products Used for Glaucoma Treatment and Their Mechanism of Action

In view of the role played by oxidative stress and neuroinflammation in glaucoma, the use of antioxidants may represent an alternative approach for glaucoma treatment. Currently, the mainstay of glaucoma treatment is the reduction in IOP, using IOP-lowering eye drops [89]. Other glaucoma treatments include laser trabeculoplasty and cyclodestruction, or surgical trabeculectomy, trabeculotomy, deep sclerectomy and viscocanalostomy, based on the European Glaucoma Society guidelines [90]. However, even when the IOP normalizes, the disease still progresses and affects visual function in some patients.

There has been significant research interest in complementary and alternative medicine (CAM), and it has been widely used in the treatment of glaucoma. In a survey involving a total of 1516 glaucoma patients in Canada, 10% of patients used CAM therapy specifically for glaucoma, and half of them believed that the treatments were beneficial [91]. Other recent surveys reported the prevalence of CAM usage to be 22% in Saudi Arabia and 67% in Palestine among eye patients [92,93]. The present review highlights recent studies on various CAMs used for the treatment of glaucoma.

### 4.1. Gingko biloba *L.*

*Ginkgo biloba* L. (GB) belongs to the Ginkgoceae family, and its leaves and seeds have been used for medicinal purposes for centuries [94]. With more than 70 different flavonoids having been identified in GB, it has been suggested to have broad-spectrum free radical scavenging activities [95]. Indeed, treatment with GB extract was able to increase the survival of a rat RGC line, following exposure to oxidative stress induced by hydrogen peroxide (H_2_O_2_) [96]. Furthermore, POAG patients treated with 120 mg of GB extract daily for at least 6 months demonstrated a lower rate of single-stranded DNA breaks in circulating leukocytes, indicating reduced oxidative stress [97]. 

Numerous clinical trials have also demonstrated that GB extract supplementation slows the progression of visual field damage and improves visual function in NTG patients [98,99]. However, Shim et al. [99] demonstrated that supplementation with 40 mg of GB extract, three times per day, showed no effect on the mean defect or contrast sensitivity in NTG patients, compared to those receiving placebo. Based on the vascular hypothesis of glaucoma pathogenesis, NTG patients receiving 80 mg GB extract tablets, twice a day for four weeks, showed a significant increase in ocular blood flow, volume and velocity, in comparison to the placebo group [100]. Furthermore, GB supplementation increased the radial peripapillary capillary vascular density in healthy subjects who received a 120 mg GB extract capsule daily for 4 weeks [101]. Table 2 summarizes clinical trials of natural products used for glaucoma treatment.

In animal studies, intraperitoneal injections of GB extract administered after optic nerve injury in rats were associated with a higher survival rate of RGCs [96,102]. This could be due to the anti-apoptosis property of GB, as demonstrated by the inhibition of apoptosis of RGCs via the modulation of mitogen-activated protein kinase (MAPK) signaling pathways, in the adult rat optic nerve injury model, following the retrobulbar injection of diterpene ginkgolides meglumine injection (DGMI, made from GB extracts, including ginkgolides A, B and K) [103]. Mechanistically, DGMI could inhibit cell apoptosis by inhibiting p38, JNK and Erk1/2 activation [103]. Additionally, GB extract-derived procyanidin B2 and rutin were shown to be able to protect human retinal pigment epithelial cells subjected to tert-butyl hydroperoxide-induced oxidative stress by modulating nuclear factor erythroid 2-related factor (Nrf)-2 and Erk1/2 signaling [104]. Another study proposed that P53, Bax, Bcl-2 and caspase-3/-9 could be considered as the core targets for GB extract against apoptosis in H_2_O_2_-treated RGCs [105]. A summary of preclinical studies of natural products used for glaucoma treatment is provided in Table 3.

### 4.2. Scutellaria baicalensis Georgi—Baicalin, Baicalein and Wogonin

*Scutellaria baicalensis* Georgi, commonly known as Baikal skullcap or Chinese skullcap, is a widely used Chinese medicinal herb [106]. *S. baicalensis* extract and its three major active flavonoids, namely, baicalin, baicalein and wogonin showed low cytotoxicity and possessed neuroprotective, antioxidant, anti-inflammatory and anti-cancer properties [106,107,108].

Intragastric administration of 200 mg/kg of baicalein for 28 days significantly reduced IOP in a rat model of chronic OHT [109]. The decreased thickness of the RGC complex and the reduced nucleus of the RGC layer mediated by OHT were significantly ameliorated by baicalein treatment and associated with reduced apoptosis of RGCs by upregulating the expression of the anti-apoptotic protein Bcl-2 [109]. Additionally, baicalein protects RGCs against retinal ischemia via the downregulation of HIF-1α, matrix metalloproteinase (MMP)-9 and vascular endothelial growth factor (VEGF), and upregulation of HO-1 [110].

The intraperitoneal administration of wogonin, 10 min after the establishment of the optic nerve crush rat model, reduced the loss of RGCs and inhibited RGC apoptosis [111]. The study also demonstrated the anti-inflammatory property of wogonin in preventing TLR4-NF-κB-mediated neuroinflammation, as indicated by the reduced gliosis response, microglial activation and pro-inflammatory cytokine (TNF-α, monocyte chemoattractant protein-1 (MCP-1), iNOS, IL-6 and -1β and cyclooxygenase (COX-2)) expressions in the retina following optic nerve crush [111].

Intraperitoneal administration of baicalin increased the number of RGCs and attenuated pathological changes (indistinct layer of retinas, decrease in the thickness of the RGC layer (GCL, a retinal layer where RGCs and displaced amacrine cells reside) and RGC density) in a model of episcleral venous occlusion with cauterization to establish a mouse model of glaucoma with chronic elevated IOP [112]. Baicalin treatment also inhibited autophagy and activated PI3K/AKT signaling in glaucoma mice, as PI3K/AKT signaling was shown to restrain the apoptosis and inflammatory response of RGCs in glaucoma development [112]. Additionally, treatment with baicalin significantly increased cell survival, reduced ROS production and inhibited pro-inflammatory factor IL-1α and endothelial leucocyte adhesion molecule-1 (ELAM-1) production in cultured human TM cells exposed to H_2_O_2_ [113].

### 4.3. Coleus forskohlii (willd.) Briq.—Forskolin

*Coleus forskohlii* (willd.) Briq. is a medicinal plant indigenous to India and Southeast Asia [114]. The leaves, roots and tubers of *C. forskohlii* are a rich source of a diterpenoid called forskolin, which acts as a second messenger cyclic adenosine 3′,5′-monophosphate (cAMP) booster, via the direct stimulation of adenylate cyclase [114]. Studies have revealed that cAMP is important in regulating aqueous humor dynamics in the ciliary body and TM [115]. Indeed, a previous study has shown that forskolin perfused arterially at 30, 100 and 1000 nM caused a significant reduction in the rate of aqueous humor formation in an isolated bovine eye preparation [116]. This may explain the hypotensive effect of forskolin administration, as shown in a double-blind, randomized controlled trial where POAG patients treated with forskolin 1% *w*/*v* aqueous solution eye drops, at two drops thrice a day, for 4 weeks, showed a significant decrease in IOP [117,118].

In animal studies, a dietary combination of forskolin, homotaurine, spearmint and vitamins B1, B6 and B12 was able to protect against RGC loss in a rodent model of optic nerve injury [119] and hypertensive glaucoma [120]. Both studies demonstrated that the forskolin supplement mixture may counteract the inflammatory processes via the reduction in cytokine (iNOS, IL-6 and TNF-α) secretion, thereby leading to decreased apoptotic markers (Bax/Bcl-2 ratio and active caspase-3), finally sparing RGC death and the preservation of visual function [119,120]. However, in contrast to the clinical studies, the forskolin supplement mixture did not affect IOP elevation in glaucomatous rodents [120].

### 4.4. Erigeron breviscapus (vant.) Hand. Mazz.—Scutellarin

*Erigeron breviscapus* (vant.) Hand. Mazz. (DengZhanHua in Chinese) is a dicotyledonous plant in the Compositae chrysanthemum family found primarily in southwest China, especially in Yunnan [121]. It has been used in traditional Chinese medicine, for the prevention and treatment of cardiovascular diseases [121]. *E. breviscapus* supplements administered for 6 months showed no obvious adverse effects, with a significant decrease in the mean defect and an increase in the mean sensitivity, in POAG patients with a controlled IOP, demonstrating its partial protective effect on the visual field in glaucoma [122]. In chronic elevated IOP animal models, *E. breviscapus* oral supplements were shown to reduce IOP, improve impaired visual function, increase the RGC density and reduce RGC axonal degeneration caused by elevated IOP [123,124]. In RGCs, *E. breviscapus* extract was shown to suppress the outward potassium channel currents, which was suggested to be one of the key mechanisms behind *E. breviscapus*’s beneficial effects against glaucoma-induced RGC damage and visual impairment [125].

The flavonoid scutellarin is one of the major constituents of *E. breviscapus*. A 3-week oral scutellarin treatment ameliorated retinal thinning and visual deficits in an induced chronic OHT glaucoma model [126]. Scutellarin protected RGCs and reduced impaired retinal microglial cells by inhibiting NLRP3 inflammasome-mediated inflammatory reactions, which was associated with a reduced upregulation of apoptosis-associated speck-like protein (a caspase recruitment domain), cleaved caspase-1 and IL-18 and -1β following acute OHT [127].

### 4.5. Lycium barbarum *L.*

*Lycium barbarum* L., commonly known as goji berry or wolfberry, has been widely used in China to treat various diseases, i.e., blurry vision, abdominal pain, infertility, dry cough, fatigue, dizziness and headaches, and has been used as a potent anti-aging agent [128]. The most abundant component in goji berries is represented by carbohydrates, and isolated *L. barbarum* polysaccharides (LBPs) have been found to exert various pharmacological properties, i.e., neuroprotective, hypoglycemic, anti-cancer, immunomodulatory and antioxidant properties [129,130]. LBP supplementation has been shown to protect RGC survival and preserve retinal function in various glaucoma models, i.e., acute OHT [131,132], chronic OHT [133,134] and partial optic nerve transection [135]. In the partial optic nerve transection model, LBP pre-treatment for 7 days prior to the injury was shown to delay secondary degeneration of RGCs [136]. The study also reported LBP exerting its neuroprotective effects by inhibiting oxidative stress and the JNK/c-jun pathway, and by transiently increasing the expression of insulin-like growth factor-1, which is a known neurotrophic factor determining the survival of RGCs during the early stages of optic nerve injury [136].

LBP has been shown to protect RGCs against oxidative stress injury by inhibiting the generation of ROS and reducing the mitochondrial membrane potential following cobalt chloride (CoCl_2_)-induced hypoxia [137]. Additionally, LBP significantly promoted cell viability, reduced apoptosis and decreased cleaved caspase-3/-9 and ROS levels in human TM cells after H_2_O_2_ administration [138]. Alternatively, LBP treatment has been shown to promote M2 polarization of microglia and downregulate autophagy after partial optic nerve resection, which contributes to the delayed secondary degeneration of RGCs [139]. Other studies have also suggested that LBP provides neuroprotection to the RGCs and retina by inhibiting vascular damage, probably via the regulation of endothelin-1 (ET-1)-mediated biological effects [131,133]. In a recent study, LBP treatment also promoted blood–retinal barrier maintenance and survival of RGCs in acute OHT mice, which were mediated through the regulation of amyloid-β production and advanced glycosylation end product receptor expression [140]. Furthermore, *L. barbarum* ethanolic extracts reduced angiopoietin-like 7 protein (ANGPTL7) expression while increasing that of caveolin-1 in PC12 neuronal cells exposed to hydrostatic pressures, which was associated with decreased gene expressions of ECM proteins, i.e., MMP-2, MMP-9, collagen I and TGF-β [141]. Previous studies have indeed indicated that ANGPTL7 modulates the TM’s ECM [142] and MMP-mediated ECM turnover in the TM, which leads to a reduction in outflow resistance in the conventional outflow pathway, and to maintenance of IOP homeostasis [143].

LBP treatment significantly reduced neuronal death and glial activation in the retina following I/R injury [144,145]. Furthermore, LBP treatment was able to alleviate ischemia-induced retinal dysfunction (exhibiting greater b-wave and oscillatory potential responses) [144,146]. The antioxidant levels (glutathione, SOD and CAT) in the retina were significantly higher, while the MDA level was lower, in the submicron and blended *L. barbarum* extract-treated groups, compared to the control [146]. Further studies demonstrated that LBP exerted its neuroprotective effects via the activation of Nrf2 and an increase in HO-1 protein expression in the retina after I/R injury [145].

### 4.6. Diospyros kaki *L.*

Persimmon (*Diospyros kaki* L.), belonging to the family Ebenaceae, is a well-known fruit rich in carbohydrates, dietary fibers, vitamins, minerals, carotenoids, phenolic compounds and other bioactive phytochemicals [147]. In addition to its fruit, persimmon’s leaves are also rich in flavonoids that exhibit antioxidant properties [148]. Pre-treatment of RGCs exposed to excessive oxidative stress and excitotoxicity with an ethanolic extract of persimmon leaves (EEDK) increased cell viability in a concentration-dependent manner [149]. Further studies revealed that the neuroprotective effect of EEDK was associated with decreased levels of apoptotic markers, i.e., poly (ADP-ribose) polymerase, p53 and cleaved caspase-3, and increased expression levels of antioxidant enzymes, i.e., SOD, GPX and glutathione S-transferase [149]. The same study demonstrated that EEDK treatment protects the retina and RGCs in a partial optic nerve crush mouse model [149]. Additionally, EEDK was also shown to reduce elevated IOP in a glaucoma mouse model, by regulating the soluble guanylate cyclase α-1 (sGCα-1, a primary regulator of vascular hypertension) signal [150].

### 4.7. Tripterygium wilfordii Hook F.—Triptolide and Celastrol

*Tripterygium wilfordii* Hook F., commonly known as thunder god vine, is a traditional Chinese medicine widely used to treat autoimmune and inflammatory diseases including rheumatoid arthritis, systemic lupus erythematosus and dermatomyositis [151]. Triptolide and celastrol are the predominant active phytochemicals isolated from this plant, which exhibit similar pharmacological activities, i.e., anti-cancer, anti-inflammatory, immunosuppressive, anti-obesity and anti-diabetic activities [152]. Triptolide treatment improved RGC survival via the inhibition of microglial activation in glaucoma models [153,154,155]. Additionally, triptolide treatment inhibited the expression of TNF-α and the nuclear translocation of NF-κB in an optic nerve crush model, suggesting that the neuroprotective effect of triptolide was attributed, partly, to its anti-inflammatory property [155]. Similarly, celastrol treatment also improved RGC survival in glaucoma models [156,157].

### 4.8. Crocus sativus *L.*—Crocetin and Crocin

Saffron (the dried stigma of *Crocus sativus* L.) is a spice that is widely used in food preparation, as a flavoring and coloring agent [158]. Referred to as the ‘golden spice’, saffron is the highest-priced aromatic medicinal plant in the world, with numerous pharmacological properties such as anti-cancer, anti-diabetic, anti-inflammatory, antioxidant, immunomodulatory, antifungal and antimicrobial properties [158]. Oral administration of saffron extract was shown to decrease microglial numbers and their activation following increased IOP, and this led to the prevention of RGC death [159]. A randomized interventional pilot study revealed that 30 mg/day saffron supplementation significantly reduced IOP in POAG patients, after 3 weeks of treatment [160].

More than 150 chemical compounds have been extracted from saffron, with crocin and crocetin being the two major active ingredients [161]. Intraperitoneal treatment with crocin can inhibit I/R-induced RGC death, and the effect of crocin may be mediated, partly, by its antioxidant action through the ERK pathway [162], or activation of the PI3K/AKT signaling pathway [163]. Additionally, crocin protects RGCs against H_2_O_2_-induced damage by reducing ROS production and activating NF-κB [164]. Similarly, crocetin, an aglycone of crocin, prevented cell loss and apoptosis in the GCL in mice following NMDA- [165] and I/R-induced retinal damage [166].

### 4.9. Curcuma longa *L.*—Curcumin

Curcumin is a yellow pigment and an active component of the rhizome of *Curcuma longa* L., or turmeric [167]. It is known to possess antioxidant, anti-inflammatory, anti-cancer, anti-arthritis, anti-asthmatic, antimicrobial, antiviral and antifungal properties [167,168]. Considering that curcumin is a powerful antioxidant natural compound, it may represent another potential treatment to alleviate oxidative stress in glaucoma. Using an elevated IOP rodent model, curcumin treatment decreased the intracellular level of ROS and alleviated RGC apoptosis induced by oxidative stress [169]. In the same study, it was also observed that curcumin inhibited pro-apoptotic factors, such as caspase-3 and Bax, and upregulated the anti-apoptotic factor Bcl-2 [169]. In an ex vivo optic nerve injury model, thinning of retinal layers, especially the GCL, and strong RGC apoptosis were observed after 24 h post-injury, which correlated with a time-dependent increase in caspase-3 and -9 and pro-apoptotic marker levels, and a powerful activation of the JNK, c-Jun and ERK signaling (MAPK) pathways [170]. Curcumin prevented alterations in the apoptotic cascade and MAPK pathways, preserving RGC survival and retinal thickness [170]. In another experimental study in a rat retinal I/R injury model, curcumin supplementation in the diet for 2 days before I/R was able to protect the retina from ischemic injury [171]. Additionally, curcumin pre-treatment inhibited I/R-induced degeneration of retinal capillaries, which may occur through its inhibitory effects on injury-induced activation of NF-κB and signal transducer and activator of transcription 3 (STAT3), and on overexpression of MCP-1, a chemokine involved in the inflammatory response via recruitment of monocytes to injury sites [172].

Studies using TM cells exposed to H_2_O_2_-induced oxidative stress as an in vitro model observed that pre-treatment with curcumin reduced the production of intracellular ROS in a dose-dependent manner [173,174]. Curcumin alleviated oxidative stress-induced pro-inflammatory factors such as IL-1a, -6 and -8 and ELAM-1 and inhibited the apoptosis of TM cells [173]. Curcumin has also been shown to protect TM cells against oxidative stress and apoptosis via the Nrf2-keap1 pathway [174].

### 4.10. Camellia sinensis *(L.)* Kuntze—Epigallocatechin-3-Gallate

*Camellia sinensis* (L.) Kuntze, commonly known as green tea, is consumed as a beverage and is popular in China and Japan [175]. Green tea extract treatment administered orally to retinal I/R injury rats showed a higher number of surviving RGCs, and less apoptotic RGCs were observed [176]. Green tea extract treatment also reduced the increased protein expression (i.e., of apoptotic markers (activated caspase-3 and -8) and inflammation-related proteins (Toll-like receptor 4 (TLR4), IL-1β and TNF-α)) and p38 phosphorylation caused by the ischemic injury [176]. Additionally, green tea extract treatment led to suppression of activated microglia, astrocytes and Müller cells following lipopolysaccharide (LPS)-induced retinal inflammation in rats [177]. The green tea anti-inflammatory effects were associated with a reduction in the phosphorylation of STAT3 and NF-κB in the retina [177].

The major polyphenolic compounds contained in green tea are catechins, which include epigallocatechin-3-gallate (EGCG), which is also a powerful antioxidant, anti-angiogenic and anticarcinogenic agent [175,178]. EGCG treatment was shown to preserve the RGC density in acute [179] and chronic elevated IOP rats [180], an optic nerve crush rat model [181], a retinal I/R injury rabbit model [182] and NMDA-induced excitotoxicity in rats [183]. Zhang et al. [179] reported that EGCG treatment significantly decreased inflammation-associated cytokine levels (IL-4, -6, -1β and -13, TNF-α and IFN-γ), and the proliferation rate of T lymphocytes. Furthermore, EGCG treatment inhibited the increase in the phosphorylation of nuclear factor of kappa light polypeptide gene enhancer in B cells inhibitor, alpha (IκBα) and p65, leading to the suppression of NF-κB signaling pathway activation [179].

### 4.11. Panax ginseng—Ginsenoside

*Panax ginseng*, in the family Araliaceae, is considered as one of the most frequently employed medicinal herbs and functional foods [184,185]. In a randomized, placebo-controlled, crossover study, daily consumption of 3 g of Korean red ginseng (KRG) for 4 weeks was shown to improve daytime contrast sensitivity and ocular pain in glaucoma patients [186]. Following 8 weeks of KRG supplementation, glaucoma patients showed significant improvement in their tear film stability and total Ocular Surface Disease Index score, suggesting KRG improved dry eye syndrome in glaucoma patients [187]. Additionally, OAG patients receiving 1.5 g of KRG, orally 3 times daily for 12 weeks, showed significant improvement in the retinal peripapillary blood flow in the temporal peripapillary region [188].

Ginseng contains numerous phytochemicals such as ginsenoside (triterpenoid saponin), phenols and acidic polysaccharides [189]. These phytochemicals have been shown to protect RGCs. Total *Panax notoginseng* saponin treatment increased RGC survival and inhibited the cell apoptosis pathway induced by an optic nerve crush rat model [190]. Similarly, ginsenoside Rg1 treatment was able to reduce RGC damage in an ultrasound-targeted microbubble optic nerve damage rabbit model [191]. Furthermore, ginsenoside Rb1 protects RGCs against apoptosis caused by CoCl_2_-induced hypoxia and H_2_O_2_-induced oxidative stress [192].

### 4.12. Cannabis sativa—Cannabinoids

*Cannabis sativa,* commonly known as marijuana, is one of the most used psychoactive substances in the world [193]. The *C. sativa* plant contains more than 60 lipid-based cannabinoids, which are the signaling molecules of the endocannabinoid system; these include Δ-9-tetrahydrocannabinol (Δ^9^-THC), Δ-8-tetrahydrocannabinol (Δ^8^-THC), cannabidiol and cannabinol [194]. A reduction in IOP was observed in glaucoma patients associated with tachycardia, within the first 30 min after marijuana inhalation, with the duration of action limited to 4 h [195]. Similarly, Δ^9^-THC inhalation reduced IOP significantly from baseline in healthy adult subjects, detected from 40 min post-treatment and lasting up to 4 h [196].

In animal studies, a topically applied 2% Δ^9^-THC ophthalmic solution was shown to reduce IOP in clinically normal dogs [197]. To prolong the IOP reduction duration, the use of Δ^9^-THC-valine-hemisuccinate nanoemulsions, which help to increase absorption, produced a greater drop in IOP, compared to latanoprost and timolol in normal rabbits [198]. Similarly, a submicron emulsion of Δ^8^-THC treatment to normal and OHT rabbits also demonstrated a reduced IOP [199]. The IOP-lowering and RGC neuroprotective effects of cannabinoids have been shown to be mediated by CB1 cannabinoid receptors [200,201].

### 4.13. Anthocyanins

Anthocyanins, considered as flavonoids, are blue, red or purple pigments commonly found in the flowers, fruits and tubers of many plants [202]. Hence, the primary sources of anthocyanins are found in berries, currants, grapes and some tropical fruits [202]. Studies have demonstrated that anthocyanins provide numerous health benefits such as antioxidative and neuroprotective properties, prevention of cardiovascular diseases, anti-angiogenesis, anti-cancer, anti-diabetic, anti-obesity and antimicrobial activities and improved visual health [202,203].

OAG patients receiving supplementation of 50 mg of black currant anthocyanins daily for 24 months also showed a reduced IOP and improved visual field damage progression [204]. Black currant anthocyanin supplementation also enhanced blood flow to the ONH and its surrounding retina in OAG patients, with no changes in systemic conditions such as blood pressure and pulse rates observed [204,205]. Black currant anthocyanin supplementation also normalized the abnormal serum concentration levels of ET-1 in OAG patients, suggesting that anthocyanins possibly affect the ET-1 receptor functions such as pharmacological reactivity and hypersensitivity [206].

The natural anthocyanins delphinidin, luteolinidin and peonidin were shown to be non-toxic to human retinal pigment epithelial (ARPE19) and RGC-5 cells, with luteolinidin and peonidin increasing the survival rates of the RGC-5 cells following exposure to H_2_O_2_ [207]. Administration of oral bilberry extracts rich in anthocyanins was shown to suppress RGC death following an optic nerve injury mouse model [208]. Bilberry extract administration increased chaperone molecule (Grp78 and Grp94) protein levels, an effect which may underlie the neuroprotective effect of bilberry extract after optic nerve crush [208]. In a model of light-induced retinal damage in pigmented rabbits, administration of bilberry anthocyanin extract at dosages of 250 and 500 mg/kg/day for 7 days significantly inhibited retinal dysfunction, as evidenced by the increased retinal outer nuclear layer thicknesses and lengths of the outer segments of the photoreceptor cells, compared to untreated rabbits with retinal degeneration [209]. Additionally, anthocyanin treatment attenuated the changes caused by light to the apoptotic proteins Bax, Bcl-2 and caspase-3 and increased the antioxidant enzyme levels (SOD, GPX and CAT), but it decreased the MDA level in the retinal cells [209].

### 4.14. Resveratrol

Resveratrol (trans-3,4′,5-trihydroxystilbene) is a polyphenol found in berries, grapes, pomegranates and red wine [210]. It has been reported to possess a wide range of pharmacological effects, including cardioprotection, neuroprotection and anti-diabetic activity, due to its potent antioxidant and anti-inflammatory properties [210]. Resveratrol has been reported to increase oxidative stress markers, and the nitric oxide level in human glaucomatous TM cells, possibly by increasing endothelial nitric oxide synthase (eNOS) expression and reducing inducible NOS expressions [211]. In experimental glaucoma models, resveratrol treatment was shown to reduce RGC death [212,213]. Cao et al. [213] further demonstrated that intravitreal administration of resveratrol rescued RGCs by the decreased ROS generation in RGCs of a microbead-induced high-IOP mouse model. These studies support the antioxidant properties of resveratrol, which could be beneficial in glaucoma treatment.

Resveratrol protects RGC-5 cells against H_2_O_2_-induced apoptosis, by reversing H_2_O_2_-induced increased expressions of cleaved caspase-3/-9, production of ROS and the expressions of *p*-p38, *p*-ERK and *p*-JNK, proposing that resveratrol suppresses MAPK cascades to exert its neuroprotective effects in RGCs [214]. Additionally, resveratrol also mitigates retinal I/R injury-induced RGC loss, glial activation and retinal function impairment by inhibiting the HIF-1a/VEGF and p38/p53 pathways while activating the PI3K/AKT pathway [215,216,217].

In both the chronic OHT rat model and RGC-5 cells incubated under elevated pressure, RGCs showed apoptosis and mitochondrial dysfunction [218]. Resveratrol treatment improved the expression of proteins involved in mitochondrial biogenesis and dynamics, i.e., AMPK, Nrf-1, mitochondrial transcription factor A (Tfam), mitofusin 2 (mfn-2) and optic atrophy 1 (OPA1), which led to a decrease in RGC apoptosis, mitochondrial membrane potential depolarization and ROS generation [218,219]. Another recent study identified a potential mechanism involving the protective role of resveratrol in preventing ONH astrocyte dysfunction and degeneration, which would enable the astrocytes to continue providing structural and nutrient support to the optic nerve [220].

### 4.15. Hesperidin

Hesperidin is a flavanone commonly found in citrus fruits such as oranges, tangerines, lemons and grapefruits, known for its anti-inflammatory, antioxidant and anticarcinogenic properties [221]. The antioxidant profile of a novel supplement containing hesperidin, and two other food-derived antioxidants, i.e., crocetin and *Tamarindus indica* (tamarind), was assessed in a prospective, single-arm design trial involving 30 NTG patients receiving the supplements for 8 weeks [222]. In patients with relatively high oxidative stress, the supplement significantly reduced the urinary 8-hydroxy-2′-deoxyguanosine (8-OHdG; a marker of oxidative DNA damage) level, and the biological antioxidant potential was also significantly elevated [222].

In an animal study, a single dose of oral hesperidin pre-treatment (25, 50 and 100 mg/kg) significantly reduced the increased IOP level in dextrose- and prednisolone acetate-induced OHT rats [223]. Additionally, hesperidin treatment increased the glutathione level in the aqueous humor and reduced morphological alteration in the ciliary bodies caused by elevated IOP [223]. Furthermore, hesperidin treatment ameliorated NMDA-induced retinal injury by suppressing oxidative stress [224] and excessive calpain activation [225] while also alleviating hypobaric hypoxia-induced retinal impairment through the activation of the Nrf2/HO-1 pathway [226]. 

### 4.16. Caffeine

Caffeine (1, 3, 7-trimethylxanthine) is a natural alkaloid commonly consumed through coffee, tea, carbonated soft drinks, energy drinks, chocolate and other cocoa-containing foods [227]. Caffeine acts as a central nervous system stimulant through its A_1_ and A_2a_ adenosine receptor antagonist properties [227]. The effect of caffeine consumption on IOP was found to be controversial in the literature. Tran et al. [228] demonstrated a reduced IOP following 45 and 60 min consumption of caffeine in POAG patients, when compared to the water-drinking group. However, another study reported that 1% caffeine eye drops administered daily for a week showed no effect on IOP in POAG patients [229]. In contrast, healthy individuals receiving a single dose of a 4 mg/kg caffeine capsule showed an increase in IOP, with low-caffeine consumers reporting a more abrupt IOP increase compared to the high-caffeine consumers [230]. Further studies suggested the increase in IOP was associated with a reduction in the anterior chamber angle, which led to resistance to aqueous humor outflow [231]. Recent cross-sectional studies showed caffeine consumption was weakly associated with a lower IOP but was not associated with a decreased risk of developing glaucoma [232,233]. An in vivo study demonstrated a reduced IOP and prevention of loss of RGCs in the caffeine-drinking animals following laser-induced OHT in experimental rats [234]. However, the same study also reported that caffeine treatment did not ameliorate OHT-induced impairment in the RGC retrograde transport, although caffeine treatment appeared to partially attenuate axonal degeneration of the optic nerve induced by OHT [234]. Interestingly, caffeine drinking led to increased microglia reactivity, inflammatory response (IL-1β and TNF mRNA levels) and cell death following 24 h post-I/R injury in a mouse model, which were then reduced at day 7 post-injury [235]. Additionally, caffeine was shown to preserve the integrity of the blood–retinal barrier in LPS-treated ARPE19 cells, which can be considered as a new strategy to treat retinal degenerative diseases [236].

### 4.17. Coenzyme Q10

Coenzyme Q10 (CoQ10), or ubiquinone-10, is a natural lipophilic vitamin-like molecule with antioxidant and anti-inflammatory properties and is involved in the production and control of cellular bioenergy, pyrimidine synthesis, physicochemical properties of cellular membranes and gene expression [237,238]. It is predominantly found in animal organs (kidney, liver and heart) and is also present in meat, fish, soy oil and peanuts [238].

Treatment with CoQ10, either topically applied or supplemented in the diet, was shown to promote RGC survival by inhibition of RGC apoptosis in glaucoma models [239,240,241]. CoQ10 treatment has also been shown to inhibit glaucomatous mitochondrial alteration by the preservation of the mtDNA content and Tfam/oxidative phosphorylation (OXPHOS) complex IV protein expressions [239,240]. Furthermore, CoQ10 treatment inhibited the activation of astrocytes and microglial cells in the retina [239,240]. In a clinical study, CoQ10 and vitamin E eye drop administration in POAG patients for 12 months showed a beneficial effect on the inner retinal function (PERG improvement), with a consequent enhancement of the visual cortical responses (VEP improvement) [242]. Additionally, CoQ10 and vitamin E topical treatment increased RGC numbers, inhibited apoptosis and activated astrocytes and microglial cells in a mechanical optic nerve injury rat model [243].

### 4.18. Vitamins

A cross-sectional study involving a total of 2912 participants in the United States 2005–2006 National Health and Nutrition Examination Survey reported that supplementary consumption and serum levels of vitamins A and E were not associated with glaucoma prevalence [244]. A meta-analysis did not find an association between serum vitamin B_6_, vitamin B_12_ and vitamin D levels and different types of glaucoma [245]. Another recent systematic review concluded that blood levels of vitamins (A, B complex, C, D and E) did not demonstrate an association with OAG as well [246]. However, the same study reported that dietary intake of vitamins A and C showed a beneficial association with OAG [246].

The nicotinamide adenine dinucleotide (NAD^+^, an important metabolite for mitochondrial metabolism and oxidative stress protection) level in the retina of D2-*Gpnmb*^+^ mice decreased with age [247]. Oral administration of vitamin B_3_ (nicotinamide, precursor of NAD^+^) was protective as both prophylaxis and an intervention of glaucoma, as shown by the reduced incidence of optic nerve degeneration, prevention of RGC soma and axonal loss and retinal nerve fiber layer thinning and preserved visual function [247,248]. In a crossover, randomized clinical trial involving 57 glaucoma patients, oral vitamin B_3_ supplementation for 6 weeks at 1.5 g/day, then for 6 weeks at 3.0 g/day, improved RGC function, but without affecting the IOP and RNFL thickness [249].

**Table 2:** Clinical trials evaluating natural products for glaucoma treatment.

Natural Products	Subjects	Treatment Regime	Clinical Findings	References
*Ginkgo biloba*	POAG patients	120 mg GB extract, 1 tablet daily, 6 months	Lower rate of single-stranded DNA breaks in circulating leukocytes (vs. untreated patients, *p* < 0.001)	[97]
	NTG patients	80 mg GB extract, 2 tablets daily, 4 years	No effect on IOP (vs. pre-treatment, *p* = 0.509) Slowed visual field damage progression (*p* < 0.001)	[98]
	NTG patients	80 mg GB extract, 2 tablets daily, 2 years	Improved HVF deviation (vs. untreated patients, *p* = 0.002)	[99]
	NTG patients	80 mg GB extract, 2 tablets daily, 4 weeks	Increased ocular blood flow, volume and velocity (vs. placebo-treated patients, *p* < 0.03)	[100]
	Healthy subjects	120 mg GB extract, 1 tablet daily, 4 weeks	Increased radial peripapillary capillary vascular density (vs. pre-treatment, *p* < 0.021)	[101]
Forskolin	POAG patients	Forskolin 1% *w*/*v* aqueous solution eye drops, 2 drops thrice a day, 4 weeks	Reduced IOP (vs. timolol-treated patients, *p* < 0.05) No adverse events	[117]
*Erigeron breviscapus*	POAG patients	*E. breviscapus* extract, 2 tablets, 3 times daily, 6 months	No obvious adverse effects Decreased mean defect (vs. pre-treatment, *p* < 0.01) Increased mean sensitivity (*p* < 0.01)	[122]
Saffron	POAG patients	Aqueous saffron extract, 30 mg daily, 4 weeks	Reduced IOP (vs. pre-treatment, *p* = 0.0046) No obvious adverse effects	[160]
Ginseng	Glaucoma patients	Korean red ginseng, 3 g daily, 4 weeks	Improved daytime contrast sensitivity (vs. pre-treatment, *p* = 0.004) and ocular pain (*p* < 0.001)	[186]
	Glaucoma patients	Korean red ginseng, 3 g daily, 8 weeks	Improved tear film stability and total OSDI score (vs. placebo-treated patients, *p* < 0.01)	[187]
	OAG patients	Korean red ginseng, 1.5 g, 3 times daily, 12 weeks	Improved retinal peripapillary blood flow in the temporal peripapillary region (vs. pre-treatment, *p* = 0.005) No changes in blood pressure, heart rate, IOP and visual field indices	[188]
Marijuana	Glaucoma patients	Marijuana smoking, single dose	Reduced IOP (vs. placebo-treated patients, *p* value not defined) Increased heart rate	[195]
	Healthy subjects	Marijuana smoking, single dose	Reduced IOP (vs. pre-treatment, *p* < 0.01) No effect on systemic blood pressure	[196]
Anthocyanins	NTG patients	60 mg, 2 tablets daily, 2 years	Improved best-corrected visual acuity (vs. untreated patients, *p* = 0.008), and HVF deviation (*p* = 0.001)	[99]
	OAG patients	50 mg black currant anthocyanins daily, 2 years	Increased ocular blood flows (vs. placebo-treated patients, *p* = 0.01) Improved visual field damage progression (*p* = 0.039)	[204]
	OAG patients	50 mg black currant anthocyanins daily, 24 months	Reduced IOP (vs. pre-treatment, *p* = 0.027) Improved HVF deviation (*p* = 0.017) No changes in systemic blood pressure or pulse rates	[205]
	OAG patients	50 mg black currant anthocyanins daily, 24 months	Normalized serum ET-1 concentrations (vs. healthy subjects, *p* < 0.05) No changes in advanced oxidation protein products, and antioxidative activities	[206]
Hesperidin, crocetin and *Tamarindus indica*	NTG patients	Food supplement containing hesperidin (50 mg), crocetin (7.5 mg) and *T. indica* (25 mg), 4 tablets twice a day, 8 weeks	Reduced 8-OHdG level in high-oxidative stress patients (vs. pre-treatment, *p* < 0.01) Elevated BAP in high-oxidative stress patients (*p* = 0.03)	[222]
Caffeine	POAG patients	Coffee containing 1.3% caffeine (104 mg caffeine), single dose	Reduced IOP (vs. water-drinking patients, *p* = 0.012) Reduced IOP fluctuation (*p* = 0.013)	[228]
	POAG patients	1% caffeine eye drop, thrice a day, 1 week	No effect on IOP (vs. pre-treatment, *p* > 0.05)	[229]
	Healthy subjects	Caffeine capsule, 4 mg/kg, single dose	Increased IOP (vs. pre-treatment, *p* < 0.05)	[230]
	Healthy subjects	Caffeine capsule, 4 mg/kg, single dose	Increased IOP (vs. placebo-treated subjects, *p* < 0.05) Reduced anterior chamber angle (*p* < 0.05)	[231]
Coenzyme Q10	POAG patients	CoQ10 and vitamin E eye drop, 2 drops daily, 12 months	Decreased ERG P50 and VEP P100 implicit times (vs. pre-treatment, *p* < 0.01) Increased PERG P50-N95 and VEP N75-P100 amplitudes (*p* < 0.01)	[242]
Vitamin B_3_	Glaucoma patients	Vitamin B_3_ tablet, 1.5 g/day 6 weeks, followed by 3.0 g/day for 6 weeks	Improved RGC functions—PhNR Vmax (vs. placebo-treated patients, *p* = 0.03), Vmax ratio (*p* = 0.02) and visual field mean deviation (*p* = 0.02) No effect on IOP (*p* = 0.59) and RNFL thickness (*p* = 0.11)	[249]

8-OhdG, 8-hydroxydeoxyguanosine; BAP, biological antioxidant potential; ET-1, endothelin-1; HVF, Humphrey visual field; IOP, intraocular pressure; NTG, normal-tension glaucoma; OAG, open-angle glaucoma; OSDI, Ocular Surface Disease Index; PhNR, photopic negative; POAG, primary open-angle glaucoma; PERG, pattern electroretinogram; RGC, retinal ganglion cell; RNFL, retinal nerve fiber layer.

Previous studies have reported that serum vitamin D levels are significantly lower in glaucoma patients as compared to healthy subjects [250,251]. Additionally, the presence of polymorphisms in vitamin D receptors, e.g., the BsmI ‘B’ allele and TaqI ‘t’ allele, was shown to be a relevant risk factor in the development of POAG [251]. Vitamin D deficiency subjects were reported to have higher, although not significant, IOP values compared to healthy individuals [252]. Treatment with 1α,25-dihydroxyvitamin D_3_ and its analog 2-methylene-19-nor-(20S)-1α,25-dihydroxyvitamin D_3_ through eye drops reduced the IOP in normal monkeys [253]. D2 mice treated with 1 μg/kg of 1α,25-dihydroxyvitamin D_3_, intraperitoneally for 5 weeks, showed improved RGC function (increased PERG and FERG amplitudes) and reduced RGC death, compared to vehicle-treated controls [254]. Additionally, the same study also reported decreased microglial and astrocyte activation, reduced inflammatory cytokines (IL-1β and -6, IFN-γ and CCL-3) and increased expression of neuroprotective factors (BDNF, VEGF-A and PlGF) in the 1α,25-dihydroxyvitamin D_3_ treatment group [254].

Induced OHT rats fed with a vitamin E-supplemented diet showed no difference in RGC cell death, compared to normal diet-treated rats [255]. However, the same study demonstrated that dietary vitamin E deficiency aggravated RGC apoptosis following induced OHT, which was found to be related to the increased level of lipid peroxidation [255]. In contrast, both topical and systemic α-tocopherol administration preserved the RGC numbers and retinal morphology in an optic nerve crush rat model [256].

**Table 3:** Preclinical studies on natural products used for glaucoma treatment and their mechanism of action.

Natural Products	Model	RGC	IOP	Ocular Vasculation	Other Findings	References
*Ginkgo biloba*	Rat RGC cells exposed to H_2_O_2_	Increased survival rate	-	-	-	[96]
	Rat optic nerve crush model	Increased RGC density	-	-	-	[96]
	Rat optic nerve crush model	Increased survival rate	-	-	-	[102]
	Mouse RGC-5 cells exposed to H_2_O_2_	Reduced cell apoptosis	-	-	Increased antioxidant capacity (reduced T-AOC, SOD and CAT depletion)	[105]
Diterpene ginkgolides meglumine injection	Rat optic nerve injury model	Reduced cell apoptosis	-	-	Decreased conduction time of F-VEP	[103]
*Scutellaria* baicalensis—Baicalein	Rat episcleral vein cauterization-induced chronic OHT model	-	Reduced IOP	-	-	[109]
	Rat ischemic model	Reduced cell apoptosis	-	-	Upregulation of HO-1 Downregulation of HIF-1α, VEGF and MMP-9	[110]
*S. baicalensis*—Wogonin	Rat optic nerve crush model	Reduced cell apoptosis	-	-	Decreased caspase-3 activation Decreased gliosis response and microglial activation Decreased pro-inflammatory cytokine (TNF-α, MCP-1, iNOS, IL-6 and-1β and COX-2) expression	[111]
*S. baicalensis*—Baicalin	NMDA-stimulated RGC	Reduced cell apoptosis	-	-	Alleviated NMDA-induced oxidative stress (reduced ROS and MDA levels) Inhibited NMDA-induced autophagy	[112]
	Mouse episcleral venous occlusion- induced chronic OHT model	Increased RGC density Increased GCL thickness	-	-	Inhibited OHT-induced autophagy Activated PI3K/AKT signaling	[112]
Forskolin	Isolated bovine eye	-	Reduced IOP	-	Reduced peak calcium response to ATP	[116]
Forskolin, homotaurine, spearmint extract and vitamins B1, B2 and B12 mixture	Mouse optic nerve crush model	Increased RGC numbers	-	-	Reduced cytokine (iNOS and IL-6) secretion Decreased apoptotic marker (Bax/Bcl-2 ratio and active caspase-3) levels	[119]
	Rat methylcellulose-induced OHT model	Increased RGC numbers	No effect	-	Prevented the reduction in retinal function (increased PhNR amplitude, PERG amplitude and implicit time) Prevented microglial and Müller cell activation Decreased inflammatory markers (NF-κB, TNF-α and IL-6) Decreased apoptotic marker (Bax/Bcl-2 ratio and active caspase-3) levels	[120]
Sodium alginate poly (vinyl alcohol) electrospun nanofibers of forskolin	Normal rabbit	-	Reduced IOP	-	-	[257]
*Erigeron breviscapus*	Rat episcleral vein cauterization-induced OHT model	-	Reduced IOP	-	Improved visual function	[123]
	Rabbit methylcellulose-induced OHT model	Increased RGC density Increased RNFL thickness Reduced RGC axonal degeneration	-	-	-	[124]
Scutellarin	Mouse clear hydrogel-induced OHT model	-	-	-	Reduced retinal thinning Reduced visual behavioral deficits	[126]
	BV-2 cells exposed to low oxygen level	-	-	-	Increased cell viability Inhibited expression of NLRP3 Reduced the upregulation of ASC, cleaved caspase-1 and IL-18 and -1β	[127]
	Rat saline-induced acute OHT model	Increased survival rate	-	-	Reduced impaired microglial cells Inhibited NLRP3 expression Reduced upregulation of ASC, cleaved caspase-1 and IL-18 and -1β	[127]
*Lycium barbarum*	Rat argon laser photocoagulation-induced OHT model	Reduced ET-1 expression in RGCs	-	-	-	[131]
	Mouse acute OHT model	Increased RGC numbers Increased IRL thickness	-	Recovered blood vessel density in retina	Protected retinal vasculature stability (reduced IgG leakage, more continued structure of tight junctions associated with increased occludin protein level) Downregulation of RAGE, ET-1, Aβ and AGE	[131]
	Rat acute OHT model	Normalized GCL density Preserved IRL thickness	-	-	Preserved positive scotopic threshold response functions	[132]
	Rat suture implantation-induced chronic OHT model	Preserved RGCs	-	-	-	[134]
	Rat partial optic nerve transection model	-	-	-	Preserved visual function	[135]
	Rat complete and partial optic nerve transection	Delayed RGC degeneration	-	-	Increased MnSOD and IGF-1 expressions	[136]
	RGC-5 cells exposed to CoCl_2_-induced hypoxia	Reduced cell apoptosis	-	-	Inhibited ROS generation Inhibited reduction in mitochondrial membrane potential	[137]
	Human TM cells exposed to H_2_O_2_	-	-	-	Promoted cell viability Reduced apoptosis Reduced cleaved caspase-3/-9 and ROS levels	[138]
	Rat partial optic nerve transection model	Delayed secondary degeneration of RGCs	-	-	Promoted M2 polarization of microglia/macrophages Downregulated autophagy level	[139]
	PC12 cells exposed to hydrostatic pressures	-	-	-	Reduced ANGPTL7, MMP-2 and -9, collagen I and TGF-β expressions	[141]
	Mouse retinal I/R injury model	Retinal cellular organization remained normal Fewer pyknotic nuclei in GCL and INL	-	-	Reduced glial activation	[144]
	Rat retinal I/R injury model	Reduced apoptosis in GCL and INL	-	-	Increased Nrf2 nuclear accumulation Increased HO-1 expression	[145]
	Rat saline-induced acute OHT model	Downregulation of APP and RAGE expressions	-	Reverse loss of function of astrocyte endfeet around blood vessels	Reduced numbers of astrocytes and microglia Decreased glutamine toxicity in astrocytes (downregulation of glutamine synthetase)	[146]
	Rat retinal I/R injury model	-	-	-	Preserved retinal thickness Increased antioxidant levels (GSSH + GSH, SOD and CAT) Reduced MDA level	[146]
*Diospyros kaki*	Mouse microbead-induced OHT model, and D2 mouse	Reduced RGC loss	Reduced IOP	-	Increased sGCα-1 expression	[149]
	RGC-5 cells exposed to glutamate	Increased cell viability	-	-	Decreased apoptotic protein levels (poly (ADP-ribose) polymerase, p53 and cleaved caspase-3) Increased antioxidant-associated protein expression levels (SOD, GST and GPX)	[150]
	Mouse partial optic nerve crush model	Reduced RGC death	-	-	-	[150]
*T. wilfordii*—Triptolide	D2 mouse	Improved RGC survival	No effect	-	Suppressed microglia activation	[153]
	Angle photocoagulation-induced chronic glaucoma rat model	Improved RGC survival	-	-	Reduced microglia count Reduced TNF-α expression	[154]
	Mouse optic nerve crush model	Improved RGC survival	-	-	Reduced TNF-α expression Inhibited nuclear translocation of NF-κB	[155]
*T. wilfordii*—celastrol	Mouse optic nerve crush model	Improved RGC survival	-	-	Reduced TNF-α expression	[156]
	Rat trabecular laser photocoagulation model	Improved RGC survival	-	-	-	[157]
*Crocus sativus* L.	Mouse laser-induced OHT model	Prevented RGC death	-	-	Decreased microglial numbers and their activation Partially reversed downregulation of P2RY12	[159]
*C. sativus*—Crocin	Rat retinal I/R injury model	Increased RGC survival	-	-	Inhibited retinal thinning Decreased cleaved caspase-3 and *p*-ERK protein expressions Increased GSH and T-SOD activities Decreased ROS and MDA levels	[162]
	Rat retinal I/R injury model	Increased RGC survival Reduced RGC apoptosis	-	-	Upregulation of Bcl-2/Bax level Enhanced *p*-AKT levels	[163]
	RGC-5 cells exposed to H_2_O_2_	Protected RGCs from apoptosis Enhanced cell viability	-	-	Decreased LDH release Decreased ROS levels Increased ΔΨm Downregulated Bax and cytochrome c protein expressions Promoted Bcl-2 protein expression Activated NF-κB	[164]
*C. sativus*—Crocetin	Mouse NMDA-induced retinal injury model	Increased GCL density	-	-	Reduced TUNEL-positive cells Inhibited activated caspase-3/-7 Increased cleaved caspsase-3 expression	[165]
	Rat retinal I/R injury model	Increased GCL density Reduced INL thinning	-	-	Decreased TUNEL-positive cells and 8-OHdG-positive cells Decreased phosphorylation levels of p38, JNK, NF-κB and c-Jun	[166]
Curcumin	BV-2 cells exposed to H_2_O_2_	-	-	-	Increased cell viability Decreased ROS and apoptosis Downregulated caspase-3, cytochrome c and Bax Upregulated Bcl-2	[169]
	Rat episcleral vein cauterization	Prevented RGC loss	-	-	Downregulated caspase-3, cytochrome c and Bax Upregulated Bcl-2	[169]
	Ex vivo optic nerve cut model	Increased RGC survival Preserved retinal thickness			Prevented alterations in apoptotic cascades and MAPK and SUMO-1 pathways	[170]
	Rat retinal I/R injury model	-	-	-	Prevented retinal damage	[171]
	Rat retinal I/R injury model	Inhibited GCL cell loss Reduced cell apoptosis			Inhibited retinal capillary degeneration Inhibited upregulation of MCP-1, IKKα, *p*-IκBα and *p*-STAT3 (Tyr), and downregulation of β-tubulin II	[172]
	Primary porcine TM cells exposed to H_2_O_2_	-	-	-	Prevented cell death Reduced ROS production Inhibited pro-inflammatory factors (IL-6, -1α and -8 and ELAM-1) Decreased SA-β-gal activity Reduced carbonylated proteins and apoptotic cell numbers	[173]
	Primary porcine TM cells exposed to H_2_O_2_	-	-	-	Reduced ROS level Reduced apoptosis Upregulated Bcl-2 Downregulated Bax and activated caspase-3 levels Reduced Nrf2, HO-1 and NQO1 expressions Increased Keap1 expression	[174]
	Rat partial optic nerve transection model	Improved RGC density ratio	No effect	-	-	[258]
	Human TM cells exposed to H_2_O_2_	-	-	-	Reduced TNF and IL-1α and -6 expression Reduced mitochondrial ROS production Reduced cleaved caspase-3 proteins Reduced TUNEL-positive cells	[259]
Green tea	Rat retinal I/R injury model	Increased RGC numbers Reduced apoptotic RGCs	-	-	Reduced activated caspase-3 and -8, SOD2 and inflammation-related proteins expressions Reduced p38 phosphorylation Enhanced JAK phosphorylation	[176]
	Rat LPS-induced retinal inflammation model	-	-	-	Suppressed activated microglia, astrocytes and Müller glia Reduced pro-inflammatory cytokine expressions (IL-1β and -6 and TNF-α in retina and vitreous humor)	[177]
Green tea—EGCG	Rat saline-induced acute OHT model	-	-	-	Decreased inflammation-associated cytokine levels Decreased the proliferation rate of T lymphocyte cells Reduced IκBα and p65 phosphorylation	[179]
	Mouse microbead-induced OHT model	Increased RGC numbers	No effect	-	-	[180]
	Rat optic nerve crush model	Increase RGC density	-	-	Increased NF-L protein expression	[181]
	Rabbit retinal I/R injury model	Preserved organization of GCL, IPL and INL	-	-	Reduced retinal gliosis Reduced MDA level	[182]
	Rat NMDA-induced excitotoxicity model	Increased GCL cell density	-	-	-	[183]
Ginseng	Rat optic nerve crush injury model	Increased cell survival Reduced cell apoptosis	-	-	Increased Bcl-2/Bax protein ratio Decreased c-Jun, P-c-Jun and P-JNK protein expressions	[190]
	Rabbit ultrasound-targeted microbubble optic nerve injury model	Reduced RGC damage	Reduced IOP	-	Reduced oxidative stress level Reduced MDA and NO levels Increased SOD level	[191]
	RGC-5 cells exposed to CoCl_2_ or H_2_O_2_	Reduced cell apoptosis	-	-	Reduced cleaved caspase-3 and -9 expressions	[192]
Marijuana—Δ^9^-THC	Normal dogs	-	Reduced IOP	-	No effect on aqueous humor flow rate	[197]
	Normal rabbit	-	Reduced IOP	-	-	[198]
Marijuana—Δ^8^-THC	Rabbit chymotrypsin-induced OHT model	-	Reduced IOP	-	-	[199]
Marijuana	Rat retinal I/R injury model	Reduced RGC damage	-	-	-	[201]
Anthocyanins	RGC-5 cells exposed to H_2_O_2_	Increased survival rate	-	-	-	[207]
	Mouse optic nerve crush model	Increased survival rate	-	-	Increased Grp78 and Grp94 levels	[208]
Resveratrol	Glaucomatous human TM cells	-	-	-	Increased eNOS and NO levels Decreased iNOS expressions Increased IL-1α level with low dose Decreased IL-1α level with high dose	[211]
	Rat hyaluronic acid-induced chronic OHT model	Preserved RGC numbers	No effect	-	-	[212]
	Mouse microbead-induced OHT model	Preserved RGC numbers	-	-	Decreased ROS generation and acetyl-p53 expression Upregulated BDNF and TrkB expressions	[213]
	RGC-5 cells exposed to H_2_O_2_	Increased cell viability	-	-	Reduced expressions of cleaved caspase-3 and -9 Reduced ROS production Reduced loss of mitochondrial membrane potential and *p*-p38, *p*-ERK and *p*-JNK expressions Promoted SOD, CAT and GSH activities	[214]
	Mouse retinal I/R injury model	Ameliorated retinal thickness damage Increased RGC numbers	-	-	Downregulated mitochondrial apoptosis-related proteins (Bax and cleaved caspase-3) Increased Bcl-2 expression	[215]
	Mouse retinal I/R injury model	Reduced RGC loss Reduced retinal damage	-	-	Reduced TUNEL staining Reduced Bax and cleaved caspase-3 levels	[216]
	Mouse retinal I/R injury model	Reduced RGC loss	-	-	Reduced Bcl-2, Bax, caspase-3, GFAP, COX-2 and iNOS expressions	[217]
	Rat superparamagnetic iron oxide-induced chronic OHT model	No effect on GCL density Decreased cell apoptosis	No effect	-	Improved retinal morphology Improved expressions of proteins involved in mitochondrial biogenesis and dynamics	[218]
	RGC-5 cells exposed to elevated pressure	Decreased cell apoptosis	-	-	Decreased mitochondrial membrane potential depolarization Decreased ROS production Upregulated expressions of proteins involved in mitochondrial biogenesis and dynamics	[218]
	Mouse retinal I/R injury model	Decreased cell apoptosis Restored retina thickness			Increased Opa1 expression, and long Opa1 isoform-to-short Opa1 isoform ratios	[219]
	Normal rabbit	-	Reduced IOP	-	-	[260]
Hesperidin	Rat dextrose- or prednisolone acetate-induced OHT model	-	Reduced IOP	-	Increased glutathione Reduced morphological alteration in ciliary bodies	[223]
	Mouse NMDA-induced retinal injury model	-	-	-	Reduced inflammatory cytokine (TNF-α, IL-1b and -6 and MCP-1) expressions	[224]
	Mouse NMDA-induced retinal injury model	Prevented reductions in RGC markers Prevented RGC death	-	-	Reduced calpain activation, ROS generation and TNF-α gene expression Improved electrophysiological function and visual function	[225]
	Rat hypobaric hypoxia-induced retinal injury model	-	-	-	Enhanced Nrf2 and HO-1 activation Attenuated apoptotic caspase levels Reduced Bax and preserved Bcl-2 expressions Downregulated PARP1 expression Upregulated CNTF expression	[226]
Caffeine	Rat laser-induced OHT model	Increased survival rate	Reduced IOP	-	Downregulated TNF and IL-1β mRNA and protein levels Suppressed microglia activation (downregulated MHC-II, TSPO, CD11b and TREM2 expressions)	[234]
	Rat retinal I/R injury model	-	-	-	Reduced microglial activation at 7 days post-injury (reduced Iba1 and MHC-II cells; reduced TSPO and MHC-II mRNA levels) Reduced TUNEL-positive cells	[235]
	Human retinal pigment epithelial cells exposed to LPS	-	-	-	Reduced LPS-induced inflammatory cytokines (TNF-α, IL-1β and -6) Restored BDNF expression Reduced *p*-NF-κB p65 nuclear translocation Restored blood–retinal barrier (increased transepithelial electrical resistance value and ZO-1 tight junction expression)	[236]
	Mouse retinal I/R injury model	-	-	-	Increased PERG amplitude Reduced IL-6 mRNA expression Increased BDNF mRNA expression	[236]

Coenzyme Q10	Mouse retinal ischemia model	Promoted RGC survival	-	-	Prevented upregulation of SOD2 and HO-1 protein expression Blocked activation of astrocytes and microglial cells Blocked apoptosis by decreasing caspase-3 protein expression Decreased Bax protein expression Preserved Tfam protein expression	[239]
	D2-*Gpnmb*^+^ mice	Promoted RGC survival	-	-	Preserved axons in the ONH Inhibited astrocytes activation Blocked the upregulation of NR1, NR2A, SOD2 and HO1 protein expressions Decreased Bax protein expression Preserved mtDNA content and Tfam/OXPHOS complex IV protein expressions	[240]
	Rat chronic OHT model	Prevented RGC apoptosis and RGC loss	No effect	-	-	[241]
	Rat mechanic optic nerve injury model	Increased RGC numbers	-	-	Reduced activation of astroglia and microglial cells Increased Bcl-xL protein expression	[243]
Vitamin B_3_	D2-*Gpnmb*^+^ mouse	Prevented RGC loss Prevented RNFL thinning	Reduced IOP at high dose	-	Prevented the decline in NAD levels Reduced incidence of optic nerve degeneration Improved PERG amplitude Inhibited formation of dysfunctional mitochondria Decreased PARP activation Reduced DNA damage Reduced HIF-1α transcriptional induction	[247]
	D2 mouse	Increased RGC density	-	-	Increased F-PERG adaptation	[248]
Vitamin D	Normal monkeys	-	Reduced IOP	-	-	[253]
	D2 mouse	Reduced RGC death	-	-	Improved PERG and FERG amplitudes Increased neuroprotective factor (BDNF, VEGF-A and PlGF) mRNA levels Decreased microglial and astrocyte activation Decreased inflammatory cytokine (IL-1β, -6, IFN-γ and CCL-3) expressions Decreased NF-κB activation	[254]
Vitamin E	Rat episcleral vein cauterization	No effect	No effect	-	Increased serum vitamin E level	[255]
	Rat optic nerve crush model	Preserved RGC numbers	-	-	-	[256]

Δ^9^-THC, Δ-9-tetrahydrocannabinol; Aβ, amyloid beta; AGE, advanced glycation end products; ANGPTL7, angiopoietin-like protein 7; APP, amyloid precursor protein; ASC, caspase recruitment domain; Bax, Bcl-2-like protein 4; Bcl-2, B cell lymphoma 2; CAT, catalase; CNTF, ciliary neurotrophic factor; COX-2, cyclooxygenase; D2, DBA/2J; ELAM-1, endothelial leucocyte adhesion molecule-1; eNOS, endothelial nitric oxide synthase; ET-1, endothelin-1; F-VEP, flash visual evoked potentials; GCL, ganglion cell layer; GPX, glutathione peroxidase; GSH, glutathione; HIF-1α, hypoxia-inducible factor-1α; HO-1, heme oxygenase-1; IGF-1, insulin-like growth factor 1; IL, interleukin; iNOS, inducible nitric oxide synthase; IRL, insulin receptor-like; LDH, lactate dehydrogenase; LPS, lipopolysaccharide; MCP-1, monocyte chemoattractant protein-1; MDA, malondialdehyde; MHC-II, major histocompatibility complex class II; MMP, metalloproteinase; NF-κB, nuclear factor-kappa B; NF-L, neurofilament light chain; NLRP3, NOD-, LRR- and pyrin domain-containing protein 3; NMDA, *N*-methyl-d-aspartate; Nrf, nuclear factor erythroid 2-related factor; NQO1, NAD(P)H:quinone oxidoreductase; OHT; ocular hypertension; ONH, optic nerve head; OPA1, optic atrophy 1; OXPHOS, oxidative phosphorylation; PARP1, poly [ADP-ribose] polymerase 1; PERG, pattern electroretinogram; PhNR, photopic negative response; PlGF, placental growth factor; ROS, reactive oxygen species; RAGE, receptor for advanced glycation end products; RGC, retinal ganglion cell; RNFL, retinal nerve fiber layer; sGCα-1, soluble guanylate cyclase α1; SOD, superoxide dismutase; T-AOC, total antioxidant capacity colorimetric; TNF-α, tumor necrosis factor-alpha; TGF-β, transforming growth factor-beta; Tfam, mitochondrial transcription factor A; TREM2, triggering receptor expressed on myeloid cells 2; TSPO, translocator protein (18 kDa); VEGF, vascular endothelial growth factor.

## 5. Challenges for Natural Product Application in Glaucoma Treatment

The WHO has defined guidelines for evaluating the safety and efficacy of natural products, which is important to further supporting the use of CAM in the healthcare system [261]. This guideline provides general principles for both preclinical and clinical studies on evaluating herbal medicines, i.e., quality and preparation of plant materials, and general pharmacological, pharmacodynamic and toxicological analyses. Although the use of crude extracts from whole plants or a particular part of any herbal plant proves to be useful in the treatment of glaucoma, as described in this review, the identification and isolation of an active phytochemical may also be important, especially in the drug development process. Crude extracts contain a wide range of phytochemicals that may work synergistically or individually to provide a polypharmacy effect in the treatment of glaucoma [262]. Similarly, several studies have reported the use of a mixture of molecules to be effective in reducing IOP in POAG patients. Researchers may have difficulty in identifying the exact mechanism or compound responsible for such findings. For instance, oral administration of two tablets per day of a food supplement containing 150 mg of *C. forskohlii* extract (containing 15 mg forskolin), 200 mg of rutin, 0.7 mg of vitamin B1 and 0.8 mg of vitamin B2 for 30 days contributed to reducing IOP in POAG patients [263]. The same supplementation has also been shown to reduce ocular discomfort in POAG patients due to chronic use of multi-dose eye drops containing preservatives [264], and to prevent IOP spikes after neodymium:YAG laser iridotomy in patients at risk of POAG [265]. Additionally, supplementation with tablets containing *C. forskohlii* extract, homotaurine, carnosine, folic acid, vitamins of the B group and magnesium in POAG patients compensated by IOP-lowering drugs during a period of 12 months showed a significant further decrease in IOP and an improvement in the pattern electroretinogram amplitude at 6, 9 and 12 months, and foveal sensitivity at 12 months [266]. In another study, daily intake of a similar supplement for 4 months showed a decrease in IOP, improved light sensitivity and contrast sensitivity and a better quality of life in POAG patients [267]. Additionally, supplementation with French maritime pine bark/bilberry fruit extracts rich in anthocyanins to POAG patients for 4 weeks showed a reduced IOP [268].

Numerous eye drops of various classes, such as prostaglandin analogs, beta blockers, carbonic anhydrase inhibitors, adrenergic agonists, miotics and hyperosmotic agents, are often preferred over surgeries for the treatment of glaucoma [269]. One of the major issues in glaucoma treatment is patients’ noncompliance, due to improper techniques of administering eye drops [270]. Another major issue is poor drug bioavailability across the blood–retinal barrier, limited retention capacity of the cul-de-sac (usually 7–10 µL, maximum 50 µL), rapid drainage of the medication caused by gravity and washout by tearing or through the nasolacrimal duct [271]. The use of various nanoformulations such as nanoparticles, nanoemulsions and nano lipid vesicles to transport phytochemicals may be able to increase the bioavailability of the drugs to the eye. For instance, baicalein loaded in trimethyl chitosan nanoparticles showed a longer pre-ocular retention time and improved baicalein bioavailability, compared to baicalein solution [272]. Davis et al. [258] reported the use of a curcumin-loaded nanocarrier formulation using D-α-tocopherol polyethene glycol 1000 succinate nanoparticles, with each particle measuring <20 nm in diameter. In an OHT rat model, topical application of curcumin nanocarriers administered twice daily for three weeks was shown to significantly reduce RGC loss, but not in the free curcumin treatment group [258]. Additionally, the same study showed that curcumin nanocarriers protected retinal cells against CoCl_2_-induced hypoxia and glutamate-induced toxicity in vitro, by significantly increasing cell viability [258]. Similarly, a chitosan–gelatin-based hydrogel containing curcumin-loaded nanoparticles decreased the inflammation (reduced expression of TNF and IL-1α and -6, associated with downregulated mitochondrial ROS production) and apoptosis levels (reduced TUNEL-positive cells and cleaved caspase-3 protein level) of human TM cells exposed to H_2_O_2_-induced oxidative stress [259]. Apart from curcumin, co-encapsulated resveratrol and quercetin in chitosan nanoparticles, and sodium alginate-poly (vinyl alcohol) electrospun nanofibers of forskolin showed an efficient IOP reduction in adult normotensive rabbits [257,260]. These studies demonstrated that phytochemical nanoformulations hold promising results, promoting their use as an alternative to existing glaucoma eye drops in clinical practice.

Lastly, it is important to use a suitable methodology to address the objectives of a study. Numerous studies used the Bcl-2/Bax ratio to imply that the therapeutic substance influences the activation of the intrinsic apoptotic pathway in RGCs, as shown by the numerous studies which have been reviewed here. However, the concept that both Bcl-2 and Bax expressions are in a stoichiometric 1:1 balance in cells reflects the old ‘rheostat’ model of the Bcl-2 family’s protein function, a hypothetical model that was debunked over two decades ago when it was shown that a 1:1 interaction of these proteins was a laboratory artifact [273,274]. Additionally, the predominant anti-apoptotic protein expressed in the retinal cells, including the GCL, is the long form of Bcl-X (Bcl-X_L_), which was found to be 16 times more abundant than Bcl-2 [275]. Furthermore, it is even questionable whether Bcl-2 is expressed in adult RGCs and may, in fact, be limited to Müller cells in the retina [276]. Therefore, the reporting of the Bcl-2/Bax ratio may not be a suitable marker to imply apoptosis in RGCs, and instead, the changes in Bcl-X_L_ expression may correlate better with RGC apoptosis.

## 6. Conclusions

One of the most common causes of vision loss is glaucoma. Recent data have gained insight into glaucoma pathogenesis, which involves a complex interaction of LC cupping, insufficient ocular blood supply, oxidative stress and neuroinflammation. The use of natural products with antioxidant, anti-inflammatory and anti-apoptotic properties may prove to be beneficial in the treatment of glaucoma. Furthermore, natural products are easily available and are cost effective. Natural products have been shown to protect against RGC loss in in vitro and in vivo preclinical studies, as well as in clinical trials. The present review highlighted various natural products such as GBE, *L. barbarum*, *D. kaki*, *T. wilfordii*, saffron, curcumin, anthocyanin, caffeine, coenzyme Q10 and vitamins B_3_, D and E that confer neuroprotective effects on RGCs. Additionally, IOP has been shown to be reduced by treatment with marijuana, baicalein, forskolin, ginsenoside, resveratrol and hesperidin. GB, ginseng, anthocyanins and *L. barbarum* were reported to increase ocular blood flow in glaucoma. Additionally, caffeine administration has been shown to reduce IOP through its adenosine receptor antagonist properties. Although these may serve as alternative targets for glaucoma treatment other than IOP-lowering drugs, more evidence is required to warrant the recommendation of these novel targets. Admittedly, a few of these natural products have had no or limited clinical testing, restricting their potential use in the treatment of glaucoma. Nevertheless, it is important to ensure that the bioavailability and safety of these natural products are checked in well-designed randomized clinical trials to further determine their therapeutic potential in glaucoma.
